# Toward Nutritionally Sound Plant-Based Meat Analogues: Expert Consensus from a Delphi Study

**DOI:** 10.3390/foods14173068

**Published:** 2025-08-30

**Authors:** Nathalia Tarossi Locatelli, Sarah Polezi, Mariana Frazão Batista, Daniel Henrique Bandoni, Veridiana Vera de Rosso

**Affiliations:** 1 Food Labeling Observatory, Nutrition and Food Service Research Center (CPPNAC), Federal University of São Paulo (UNIFESP), Santos 11015-020, SP, Brazil; nathalia.locatelli@unifesp.br (N.T.L.); dbandoni@unifesp.br (D.H.B.); 2 Graduate Program in Nutrition, Federal University of São Paulo (UNIFESP), Santos 11015-020, SP, Brazil; sarah.polezi@unifesp.br

**Keywords:** plant-based meat analogues, Delphi study, experts, nutritional recommendations

## Abstract

Plant-based diets are increasingly recognized for their potential health benefits, reduced environmental impact, and alignment with ethical concerns related to animal welfare. Plant-based meat analogues (PBMAs), formulated using alternative vegetable protein sources, can contribute to the nutritional adequacy of such diets while supporting consumer adherence by replicating the sensory characteristics of conventional meat products. This study aimed to establish evidence-based nutritional recommendations for the formulation of nutritionally balanced PBMAs through expert consensus, using a modified Delphi method. Consensus was achieved for 12 nutritional recommendations across three stakeholder groups: (i) academic researchers; (ii) representatives from scientific societies, non-governmental organizations, civil society, and government agencies; and (iii) industry stakeholders involved in PBMA production. Recommendations focused on limiting nutrients of concern—such as sodium and saturated fats—were unanimously endorsed by all groups. Additionally, consensus was reached on recommendations emphasizing the inclusion of ingredients that ensure an adequate intake of essential nutrients, including proteins, fiber, vitamins, and minerals. Among the six proposed regulatory recommendations, three achieved consensus. The resulting set of nutritional recommendations offers a valuable framework to support the food industry in developing PBMAs that align with consumer expectations for health, nutrition, and sustainability. Moreover, these recommendations can play a pivotal role in assisting regulatory authorities in defining identity and quality standards for PBMAs.

## 1. Introduction

Accurately quantifying the global prevalence of plant-based diets presents considerable methodological challenges due to variations in definitions and data collection. Nevertheless, estimates from multiple countries suggest that approximately 9.7% of the world’s population adheres to a vegetarian or vegan dietary pattern [1]. Although Brazil ranks among the world’s leading producers, exporters, and consumers of meat [2], approximately 5% of its population identifies as vegetarian or vegan, while 34% report imposing some form of restriction on their meat consumption [3].

The adoption of diets that exclude meat and/or other animal-derived products has increased significantly across various countries. This dietary shift is influenced by multiple factors and varies according to the demographic and socio-cultural characteristics of the populations studied [4]. In general, the primary motivations for reducing or eliminating meat consumption include economic considerations, ethical and religious beliefs, nutritional and health concerns, animal welfare, and environmental sustainability. More specifically, women tend to express greater concern with nutrition and health-related factors, while younger individuals are more frequently motivated by issues related to animal welfare and environmental impacts, including greenhouse gas emissions and global warming [3,4,5]. In addition, national and international dietary guidelines recommend an increased consumption of plant-based foods and a reduction in the intake of processed meats [6,7].

However, promoting long-term, sustainable dietary changes is challenged by various social barriers. A study conducted with consumers in ten European countries identified that misconceptions rooted in popular beliefs or only partially supported by scientific evidence constitute the primary obstacles to such transitions [8]. Sensory attributes, particularly the taste of plant-based foods, along with limited availability in supermarkets and restaurants, were identified as primary barriers to their adoption. These factors were followed by nutritional concerns, such as the perception that such foods do not provide sufficient energy or strength [8] or that plant-based products are classified as ultra-processed [9,10]. Additionally, a common belief persists that plant-based diets are inadequate for supporting muscle building [11]. Economic factors, particularly product cost, also emerge as significant obstacles to dietary change, especially in low- and middle-income countries [12].

Moreover, plant-based diets encompass a wide range of dietary patterns, which can complicate the assessment of their overall nutritional quality. For instance, dietary models such as the Mediterranean diet, DASH (Dietary Approaches to Stop Hypertension), Healthy U.S.-Style Eating Pattern, Planetary Health Diet, and Nordic diet are often included under the umbrella of plant-based diets due to their emphasis on plant-derived food components. From this perspective, numerous studies have aimed to develop and apply quality indices for plant-based diets and to compare their nutritional adequacy and health outcomes with those of omnivorous dietary patterns [13,14,15,16,17,18,19,20].

Currently, there is no clear consensus in the scientific literature regarding the overall nutrient adequacy of plant-based diets. Most studies indicate that carbohydrate and dietary fiber intake among individuals following these diets aligns well with established nutritional recommendations [13]. While protein intake is generally lower than in omnivorous diets, it typically remains within recommended levels [13,21]. Findings on long-chain omega-3 fatty acid intake (e.g., EPA and DHA) are mixed, with some studies reporting adequate levels and others indicating insufficiency [13,21]. Furthermore, the intake of vitamin B12, calcium, vitamin D, and iron is frequently reported to fall below recommended values [21]. Notably, suboptimal intakes of these nutrients have also been observed in omnivorous populations [13]. Moreover, nutrient inadequacies are observed across all dietary patterns, including vegan, vegetarian, and omnivorous diets. These deficiencies are largely influenced by the specific food choices associated with each dietary pattern. Factors such as the intrinsic nutritional composition of foods, agricultural practices, post-harvest handling, industrial processing methods, and culinary preparation techniques all contribute to the variability in nutrient availability and bioaccessibility, potentially leading to pronounced inadequacies [22,23,24].

In addition to fruits, vegetables, pulses, whole grains, and nuts, vegan and vegetarian diets may be supplemented with plant-based meat analogues (PBMA). The availability of these products has expanded significantly over the past decade, with marked growth observed in the 2020s [25]. At present, a wide variety of PBMA are available on the market, designed to replicate the taste, texture, and appearance of animal-derived foods. These products exhibit diverse nutritional profiles, primarily due to the use of various plant protein sources in their formulations [10,26,27,28,29,30,31,32]. In some cases, these products may present inadequate nutritional quality relative to established dietary recommendations, characterized by elevated levels of saturated fats and sodium, and insufficient amounts of iron, vitamin B12 and proteins [23,27,31,33,34,35,36,37].

The formulation of nutritionally adequate PBMAs may impact their sensory attributes, technological properties, and production costs. Furthermore, standardized nutritional quality and identity parameters for PBMAs are generally lacking, limiting the ability of the food industry to use them as benchmarks during product development. Efforts to replicate the composition and technological processes of conventional meat products often result in plant-based analogues that fall short of consumer expectations. These factors collectively contribute to the complexity of defining an optimal composition for the development of nutritionally adequate plant-based meat analogues.

One strategy to address some of the challenges faced by the industry is the development of a guideline outlining evidence-based recommendations for the nutritional composition of PBMAs, endorsed by subject-matter experts. Such a document aims to consolidate expert opinions from diverse fields, including Food Science and Technology, and Nutrition, to support the formulation of nutritionally improved PBMAs tailored to meet consumer health needs. The development of a recommendation guide requires the establishment of consensus among the consulted experts. An effective approach for achieving this consensus is the Delphi method, which facilitates structured communication and iterative feedback among specialists [38].

The Delphi Method was developed in the early 1950s by the RAND Corporation, a research institution supported by the United States Air Force, with the aim of systematically obtaining expert consensus on complex strategic issues [39]. Beginning in the 1960s, the Delphi Method was progressively adopted across a wide range of fields [40,41]. In recent years, the Delphi Method has gained increased prominence in the fields of Food Science and Nutrition, contributing to structured debates on topics such as food safety, food security, sustainable food systems, labeling and marketing practices, and improving the development of public policies related to food and nutrition [42,43,44,45,46,47].

The topic of plant-based foods has increasingly stimulated debate within the Brazilian scientific community. Recently, a study employing the Delphi method was published with the objective of identifying and prioritizing key actions to scale up the production and consumption of plant-based meat in Brazil [12]. Experts identified the price of PBMAs as the primary barrier. Additionally, the theme of Health, Nutrition, and Safety—which included the action item ‘develop and adopt national food safety and quality standards for PBMA products’—was not ranked among the top priorities [12].

In this context, the objectives of the present Delphi study were: (i) to establish nutritional recommendations for the development of nutritionally healthy PBMAs through expert consensus; and (ii) to assess the relative importance of nutritional, sensory, technological, and regulatory attributes associated with PBMAs marketed in Brazil.

In 2024, the plant-based sector in Brazil recorded sales revenues of 1.1 billion reais (approximately US$203.7 million), representing a 14% increase compared with 2023. Notably, only 35% of the population reported having tried plant-based meat analogues, underscoring the substantial growth potential of this market [48]. The development of nutritionally healthy and environmentally sustainable plant-based products is therefore essential to address the demands of an increasingly discerning consumer segment, particularly among younger generations such as Generation Z.

## 2. Methods

This study focused on PBMAs, defined as food products formulated exclusively with plant-derived ingredients, including components sourced from algae and fungi. Its focus is the development of nutritional guidelines for products formulated with alternative proteins to meat, including items such as burgers, sausages, ham, deli slices, breaded products, among others. This study did not include dairy or egg analogue products within its scope.

It is important to note that Brazil currently lacks specific legislation governing this category of products. Regulatory standards regarding identity, quality, naming conventions, and labeling requirements have not yet been established, although they are under consideration by Brazil’s National Health Surveillance Agency (ANVISA) and the Ministry of Agriculture, Livestock and Food Supply (MAPA).

### 2.1. Expert Panel

The Delphi method typically involves a series of structured questionnaires administered in successive rounds, where participants respond individually. After each round, a summary of the group’s responses is shared with participants, fostering an indirect dialogue, and enabling the gradual construction of a collective judgment or consensus [38].

The expert panel comprised three groups of stakeholders: (i) researchers from institutes and universities engaged in the development of plant-based meat analogues (PBMAs) or conducting research in alternative proteins, protein chemistry, and/or food and nutrition; (ii) representatives from scientific societies, non-profit organizations, civil society, and government agencies; and (iii) representatives from food industry associations involved in the production of PBMAs.

Experts were selected based on the following inclusion criteria: active involvement in research, policymaking, advocacy, or industrial activities directly related to plant-based foods and protein innovation, along with demonstrable expertise in their respective fields. The selection process was carried out through three complementary approaches: (i) review of academic and professional profiles available on the Lattes Platform (a comprehensive database of Brazilian researchers’ curricula vitae); (ii) identification via professional social networks (LinkedIn, Instagram, and ResearchGate); and (iii) direct engagement with professionals at food-related fairs and exhibitions held in Brazil during 2023–2024.

Recruitment was conducted via email, which included a presentation of the study objectives, an overview of the research design, and an explanatory video. At each round of the study, additional experts were incorporated to ensure the balanced representation of all three stakeholder groups.

At all rounds of the study, information was collected regarding participants’ educational background, current professional field, years of professional experience, and their level of knowledge and interest in the research topic of plant-based meat analogues. In accordance with the premises of the Delphi method [38], expert anonymity was preserved during questionnaire responses. This approach helps to minimize potential conflicts within the group and prevents dominance by individuals, thereby encouraging more candid and unbiased responses. It also facilitates the capture of a broader range of interrelated variables and multidimensional perspectives, which are characteristic of complex issues such as the one addressed in this study.

The Ethics Committee in Research of the Federal University of São Paulo approved the present research (number 0969/2023), and all participants who took part in the study provided informed consent.

### 2.2. Delphi Methodology

The study was conducted using a modified Delphi method comprising three rounds, with a progressive inclusion of experts at each round. The basis for Delphi study was constructed employing the exhaustive list of research findings, nutritional policy recommendations [49,50,51,52,53,54,55,56,57,58], communication of food guidelines [59], dietary reference intake for essential nutrients requirements [60,61], nutrient profile model (NPM) developed for Front of Packing Labeling (FOPL) proposed in Brazil [62,63,64] and NPM proposed by Pan American Health Organization/Word Health Organization for identified ultra-processed foods [65].

#### 2.2.1. Round One

The group of experts (*n* = 10) was invited to engage in discussions to define the primary guidelines to be evaluated through the Delphi method. These discussions addressed the following key topics: (i) nomenclature of plant-based meat analogue products; (ii) desirable physicochemical, sensory, and nutritional attributes of these products; (iii) the use of ingredients and additives in their formulation; and (iv) innovations within the plant-based sector. Initially, the expert panel was invited to complete an online questionnaire (Table 1) consisting of open-ended questions, allowing them to freely express their opinions and perspectives on the topic.

#### 2.2.2. Round Two

Based on the responses collected in the Delphi first round, a comprehensive list of nutritional recommendations was developed to guide the formulation of nutritionally adequate plant-based meat analogues. In addition, recommendations about sensory, technological, and regulatory aspects were produced. The recommendations were presented to a panel of experts (*n* = 20) via an online survey, allowing them to evaluate each item in terms of relevance and adequacy. A structured Likert scale ranging from 1 to 10 was used, where 1 indicated ‘absolutely irrelevant/inadequate’ and 10 indicated ‘absolutely relevant/adequate’.

Following the completion of responses by all experts, the level of agreement for each criterion was calculated using Equation (1). Items achieving an agreement coefficient (AC) of ≥80% for both relevance and adequacy were considered to have reached consensus. Items with agreement levels below 80% were reformulated and submitted for re-evaluation in a subsequent round. Those receiving an agreement coefficient of ≤50% were excluded from further consideration. At the conclusion of this round, the research team compiled a set of recommendations for the development of nutritionally adequate plant-based meat analogues that had achieved expert consensus.

(1) $$\mathrm{AC}=\left(1-\left(\frac{\mathrm{ED}}{\mathrm{ET}}\right)\right)\;\cdot \;100$$

where ED: number of experts who disagreed with each recommendation; and ET: total number of experts who evaluated each recommendation.

#### 2.2.3. Round Three

This round was conducted through an online workshop entitled *“Development of a Nutritional Recommendation Guide for Plant-Based Meat Analogue Products”*. All experts were invited via email, resulting in 60 confirmed participants. The workshop began with a presentation of the results obtained in Project “*Evaluation of the nutritional quality of plant-based meat products based on label information and the analysis of fatty acid and amino acid profiles of products available in the Brazilian market*” [29,31]. Followed by a 60 min question-and-answer session. Subsequently, participants were asked to complete an online form containing the nutritional recommendations that had achieved consensus in the previous round.

All experts were invited to evaluate the recommendations using a real-time voting system, rating each recommendation on a Likert scale from 1 to 10 according to two criteria: (1) relevance to their respective organizations (1 = absolutely irrelevant; 10 = absolutely relevant), and (2) practical feasibility (1 = absolutely infeasible; 10 = absolutely feasible). These two criteria were referred to as ‘relevance’ and ‘feasibility and were reported using frequency distributions, means, and standard deviations. Differences in responses among the three stakeholder groups were analyzed. Consensus regarding the recommendations was established when a minimum of 60% of participants assigned a score greater than 6 for both evaluated items. Furthermore, consensus was corroborated when at least two stakeholder groups jointly provided scores exceeding 6.

### 2.3. Statistic Analysis

The data were normally distributed and presented as the mean ± standard deviation. Differences were detected using analysis of variance (ANOVA) followed by Tukey’s significant difference post hoc test, and differences were considered significant at *p* < 0.05. Statistical analysis was performed using the software Statistica 7.0 (StatSoft, Tulsa, OK, USA).

## 3. Results and Discussion

Plant-based meat analogues are already well-established in the consumer food markets of developed countries. However, their nutritional composition continues to be carefully evaluated. While plant-based diets are generally associated with health benefits [5,9,13,17,20], it is still unclear whether these benefits extend to the consumption of PBMAs. Scientific evidence on this matter remains limited. Nevertheless, progress can be made in developing recommendations for the formulation of nutritionally sound products by employing methodologies that incorporate expert opinion.

### 3.1. Panel Expert Characteristics

The participants in the expert panel represented three stakeholders with interests in the field of plant-based products. As a result, they held slightly differing perspectives on the role of nutritional composition in advancing the alternative protein sector and, consequently, in promoting the consumption of these products in Brazil. Across all rounds of the study, most experts held PhD or postdoctoral qualifications and had over 20 years of experience in the fields of Food Science and/or Nutrition. Furthermore, 70% of participants identified themselves as strong interest in the topic, given its direct relevance to their professional practice and reported possessing a high level of knowledge on the subject (Table 2). In the third round of the Delphi study, 60 experts confirmed their participation in the workshop; however, only 34 submitted responses via the online form.

### 3.2. Delphi Round One

In round one of the study the responses of the ten consulted experts regarding development and composition of plant-based meat analogues revealed divergent perspectives. Selected responses provided by the experts are presented in Table 3 to illustrate the range of perspectives on the topic. The selection was based on the relevance and depth of the contributions to the ongoing debate. Responses that lacked supporting arguments or justification were excluded from the table.

The analysis of expert responses revealed some uncertainty regarding the nutritional profile expected by Brazilian consumers for plant-based meat analogues. Several experts questioned whether consumers prioritize nutritional attributes or are more guided by sensory aspects. Nonetheless, it can be inferred from their comments that, if these products are intended to replace traditional meat, there is an implicit expectation that they should replicate key nutritional functions—particularly through lower saturated fat content and the absence of cholesterol—while potentially offering additional health benefits. Several factors may contribute to the uncertainties expressed by experts regarding the nutritional expectations of Brazilian consumers for plant-based meat analogues (PBMAs). While the absence of defined regulatory standards—such as a minimum protein content—may be one contributing factor, other important challenges include the broad variability in Dietary Reference Intake (DRI) values for protein, which depend on individual characteristics such as sex, age, body mass index (BMI), physical activity level, and physiological conditions (e.g., pregnancy, lactation, or comorbidities) [60]. Additionally, individuals following plant-based diets may encounter difficulties in meeting protein requirements through food alone, especially without dietary supplementation [13]. Although existing literature and health authority guidelines indicate that protein intake is typically sufficient when caloric needs are met or when diets are supervised by professionals [66], such ideal conditions are often not met by the general population—potentially resulting in suboptimal protein consumption.

Furthermore, concerns about meeting essential amino acid requirements may be overestimated, particularly when the daily diet includes a variety of legumes, which collectively provide complementary amino acid profiles [67]. Nonetheless, recent research conducted in Brazil indicates that achieving adequate protein and essential amino acid intake among vegan individuals often relies on the inclusion of processed and ultra-processed foods, with a notable emphasis on dietary supplements [68]. Therefore, it is important to assess the contribution of PBMAs to the adequate intake of essential amino acids [31,33], as these products not only incorporate diverse sources of plant proteins but also frequently utilize isolated and concentrated protein ingredients, which exhibit considerably higher Digestible Indispensable Amino Acid Scores (DIAAS) [69,70]. Accordingly, three nutritional recommendations related to protein and essential amino acid content were incorporated into the second round of the Delphi study.

The quantitative and qualitative lipid intake profile of individuals adhering to plant-based diets differs significantly from that of omnivores, primarily due to the predominance of unsaturated fatty acids found in vegetable oils. However, despite this favorable lipid profile, intake is typically skewed toward omega-6 (n-6) fatty acids, resulting in a high n-6/n-3 ratio—often comparable to that observed in omnivorous diets. It is well-established that n-6/n-3 ratios exceeding 10 are associated with an increased risk of cardiovascular disease [71,72]. Furthermore, the dietary intake of long-chain omega-3 fatty acids—eicosapentaenoic acid (EPA) and docosahexaenoic acid (DHA)—is generally lower in vegetarians and virtually absent in vegan diets [21].

In addition, the experts expressed confidence that Brazil’s front-of-pack nutrition labeling regulations [62,63] concerning saturated fat are adequate to limit the use of saturated fat sources commonly utilized in low- and middle-income countries, such as palm and coconut fats, particularly given the prohibition of partially hydrogenated fats [73]. Accordingly, the six recommendations proposed for the second round of the Delphi study aimed to limit total fat and saturated fat content, while promoting the inclusion of omega-3 fatty acids to ensure a higher quality lipid profile. Incorporating flaxseed oil, high-DHA canola oil, and microalgae-derived oils represents a strategic approach to fulfilling these nutritional recommendations [74,75,76,77].

Plant-based diets are generally more likely to meet recommended intakes of carbohydrates and dietary fiber [60,61], as legumes—despite their relatively high protein content (approximately 30% of dry weight)—also contain substantial amounts of carbohydrates and fiber [77,78,79,80]. Accordingly, two dietary fiber-related recommendations were included in the list of items submitted to experts in the second round of the Delphi study. They aimed at achieving fiber levels sufficient to support ‘source’ and ‘high content’ nutrient claims.

In general, sodium levels in plant-based meat analogues (PBMAs) tend to be lower than those in traditional meat products [29], likely due to the absence of curing processes. However, sodium salts are often added to PBMAs to mask the inherent bitterness of certain plant proteins. Additionally, consumer familiarity with the high sodium content of conventional meat products may affect the sensory acceptability of PBMAs, presenting a challenge for product formulation. As a result of this preference for saltier foods, individuals following plant-based diets may consume sodium in amounts that exceed recommended levels, like those adhering to omnivorous diets [13,21].

As noted by the experts in relation to saturated fat, the sodium content of PBMAs could be aligned with the thresholds established by Brazil’s front-of-pack nutrition labeling regulations [62,63]. However, for the purposes of expert evaluation, a more stringent recommendation was proposed, based on a sodium limit of 1 mg/kcal—a benchmark established by the Pan American Health Organization (PAHO)/World Health Organization (WHO) [65].

Plant-based diets—particularly vegan diets—are characterized by the absence of reliable plant sources of vitamin B12. Consequently, the intake of vitamin B12, as well as other key micronutrients such as calcium, iron, vitamin D, iodine, and zinc tends to be lower among individuals following these dietary patterns [13,17,21].

The health implications of inadequate micronutrient intake and limited bioavailability are well established and extensively documented in the scientific literature. Accordingly, two recommendations were developed based on micronutrient levels required to support ‘source’ and ‘high content’ nutrition claims and were submitted for expert evaluation. The fortification of plant-based meat analogues (PBMAs) with vitamins and minerals can serve as an effective strategy to help meet recommended intake levels of these essential nutrients.

Regarding the sensory aspects of PBMAs, the experts evaluated the products as having average quality and identified several limitations likely to affect consumer acceptability. Consequently, in the second round of the Delphi study, recommendations were formulated to emphasize the prioritization of sensory characteristics over nutritional attributes. Experts were asked to rank the relative importance of these two aspects to guide future product development.

The experts’ responses to the regulatory questions reflected concerns about the lack of federal regulations to guide the food industry and to support consumer understanding regarding the identity and quality standards of plant-based meat analogues. Accordingly, the recommendations presented in the second round of the Delphi study aimed to evaluate the experts’ understanding of the draft legislation currently under development by the Ministry of Agriculture and Livestock.

### 3.3. Delphi Round Two

The nutritional recommendations for the second round of the Delphi study e-survey were developed based on the experts’ responses, the World Health Organization’s guidelines for healthy diets, and the Dietary Reference Intakes established by the Institute of Medicine [7,49,50,51,52,53,55,56,57,58,60,61].

A total of 20 experts evaluated the relevance and adequacy of 24 recommendations using an electronic survey. Recommendations that reached an agreement level of ≥80% for both criteria were considered to have achieved consensus (Table 4). Those with agreement levels below 80% were reformulated and resubmitted for assessment in a subsequent round, whereas recommendations with ≤ 50% agreement were excluded from further consideration [38,45,46]. At this stage, all expert responses were weighted equally, regardless of stakeholder group, and collectively contributed to the overall score.

The nutritional recommendations receiving the highest agreement pertained to critical nutrients, particularly sodium and saturated fat, whose intake should be restricted in accordance with healthy dietary guidelines [55].

The recommendations related to the regulatory aspects of plant-based meat analogues (PBMAs) demonstrated consensus with respect to their definition. Specifically, it was agreed that these products should exhibit sensory properties and modes of consumption comparable to those of conventional meat products in order to be classified as analogous products for marketing purposes and the use of corresponding sales denominations. Due to the expansion of the expert panel, twenty-two recommendations, including those that had achieved consensus, were incorporated into the third round of the study.

The nutritional recommendations 3, 5, and 15 did not reach consensus at this stage of the study. These recommendations pertain to the selection of raw materials for the formulation of PBMAs that meet the requirements for essential amino acids, omega-3 fatty acids, and micronutrients—such as vitamins and minerals—that are either absent or have low bioavailability in plant-based foods. The lack of consensus on these recommendations may be attributed not only to concerns about production costs, but also to limited clarity regarding the nutritional composition of PBMAs currently available on the market. The latter issue may be addressed in the third round of the study, during which data on the nutritional composition of PBMAs will be presented to participants in a dedicated workshop.

### 3.4. Delphi Round Three

The enlargement of the expert panel influenced the consensus established in the second phase of the study. Although 60 experts confirmed their participation in the workshop (the third stage of the Delphi study), only 34 completed the e-survey. Notably, representatives from the food industry—particularly those involved in the production of PBMAs—were the most absent. This may be attributed to limited familiarity with the academic environment and the Delphi methodology. Regulatory agents were also underrepresented. In contrast, researchers from universities and research institutes constituted the most engaged group, likely due to their greater ease in understanding the methodological approach and their recognition of deliberation and consensus-building as valuable tools for addressing complex challenges [40,44,45,46,82]. Limited participant adherence is a commonly reported limitation in Delphi studies [83], as the method relies heavily on the sustained engagement and input of experts throughout the multiple rounds of the process [38].

**Figure 1:**
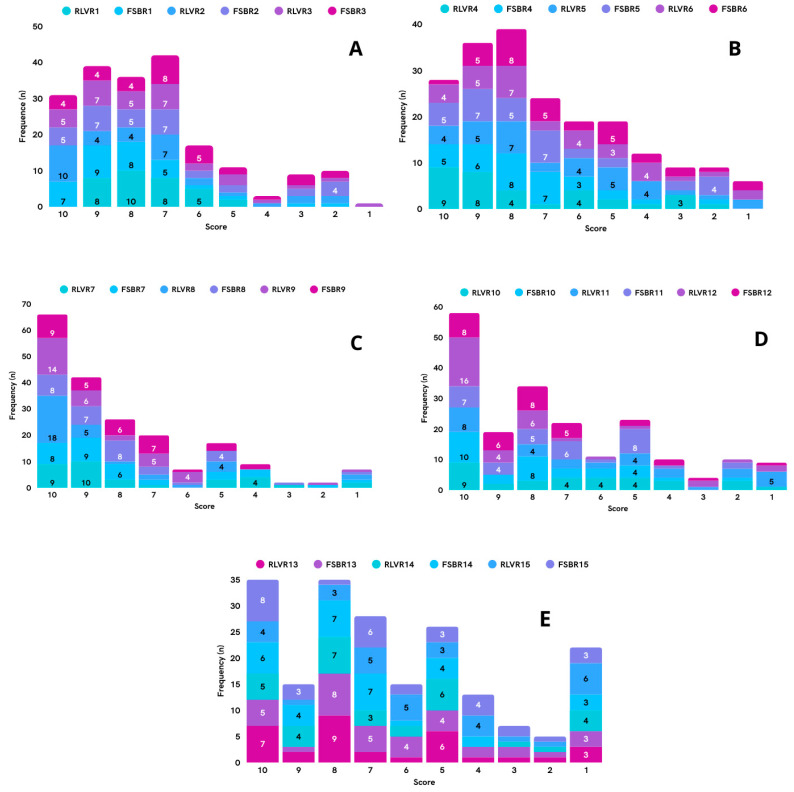
(**A**–**E**): Frequency distribution of scores assigned by experts for the relevance (RLVR) and feasibility (FSBR) criteria for nutritional recommendations. RLVR: Relevance recommendations. FSBR: Feasibility recommendations.

Total agreement was achieved based on expert assessments of relevance and feasibility, as indicated by the weighted average of these two criteria (Table 5). Figure 1 presents the frequency distribution of scores assigned by experts for the relevance and feasibility criteria for nutritional recommendations. Recommendations focused on controlling critical nutrients (e.g., total fat, saturated fat, and sodium) received higher expert ratings, whereas those related to the fortification of plant-based meat analogues (PBMAs) with vitamins and minerals were rated lower.

Recommendations 12 and 13, which address sodium levels in plant-based meat analogues, indicate that the current limit of 600 mg/100 g established by Brazilian food labeling legislation [63] is insufficient to meet the dietary sodium targets recommended by the World Health Organization (WHO) [49]. In addition, other front-of-pack nutrition labeling systems, such as the UK Nutrient Profiling Model [84] and Health Canada’s Sodium Targets [85] set more stringent limits. Consequently, the consensus recommendation of 1 mg of sodium per kilocalorie of food offers closer alignment with international sodium reduction goals.

**Figure 2:**
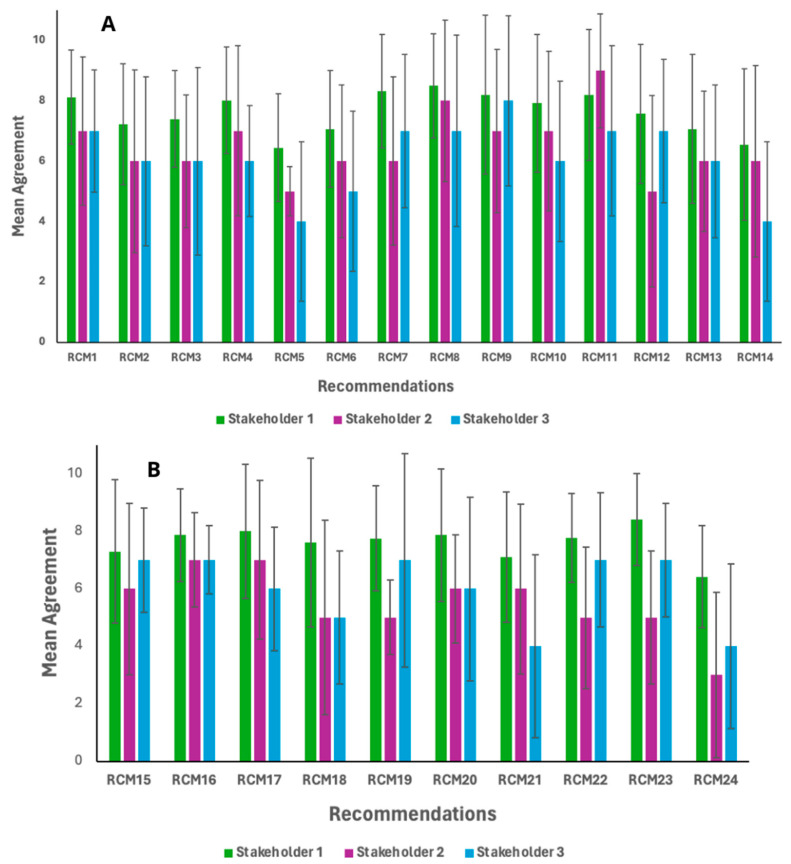
(**A**,**B**): Mean agreement between relevance and feasibility related to recommendations assigned by stakeholders. RCM: Recommendation number according to Table 5. Stakeholder 1: researchers from institutes and/or universities; Stakeholder 2: representatives from scientific societies, non-profit organizations, civil society, and government agencies; and Stakeholder 3: representatives from associations linked to the food industry that produce plant-based meat analogue products.

Consensus on the recommendations presented to the experts was determined based on two criteria: (1) a consensus threshold of over 60% agreement among the entire expert panel, and (2) a minimum average score of 6.0 (on a 1–10 scale) assigned by at least two stakeholder groups participating in the study. The adoption of a moderate consensus threshold was considered appropriate, given the anticipated divergences in the evaluation of recommendations among stakeholder groups [38]. This expectation was supported by the results presented in Figure 2, which illustrate variations in the mean agreement between stakeholder groups.

Moderate levels of consensus are common in studies where the outcomes have an indirect impact on health and well-being. Research related to food labeling and food systems, for example, may justify the use of less stringent consensus thresholds [43,83,86], whereas studies focused on clinical treatment protocols typically require higher levels of agreement due to their direct implications for health outcomes [82].

Among the nutritional recommendations evaluated—specifically those related to the protein content and amino acid profile of PBMAs—consensus was achieved for recommendations 1 and 2. However, recommendation 3, which concerned the selection of protein sources with an adequate balance of essential amino acids to support the use of a ‘source of protein’ claim, was deemed relevant and appropriate only by stakeholder group 1.

Furthermore, recommendations 5 and 14 also failed to reach consensus, as observed in the second round of the study. Even the inclusion of findings from a study that assessed the lipid profile quality of plant-based burgers available on the Brazilian market did not contribute to achieving consensus among the experts. Among the seven brands of plant-based burgers evaluated, three exhibited an omega-6/omega-3 fatty acid ratio greater than 30, with one reaching a ratio of 105 [29]. In contrast, two brands presented a ratio below 10, which aligns with the value recommended by the World Health Organization (WHO) [53,72].

Recommendations 13 and 14 differ in their proposed levels of vitamin and mineral fortification: Recommendation 13 suggests fortification at 15% of the daily value (DV), whereas Recommendation 14 proposes 30% DV. According to Brazilian food labeling regulations [63], a product containing at least 15% of the recommended daily allowance (RDA) of a given vitamin or mineral may be classified as a source of that micronutrient. For this reason, experts considered the 15% threshold (Recommendation 13) to be a more feasible target. Beyond cost considerations, it is important to note that no specific legislation currently regulates micronutrient fortification in plant-based meat analogue (PBMA) products. Consistent with findings from studies conducted in the United States, Spain, Switzerland, and Australia, the proportion of fortified PBMAs remains low, underscoring the need to establish clear guidelines for their nutritional composition [87]. As illustrated in Figure 2A, both recommendations received relatively lower scores from all stakeholder groups.

The inclusion of fortified foods or dietary supplements containing vitamin B12, vitamin D, calcium, selenium, and polyunsaturated fatty acids such as EPA and DHA were highlighted by experts in the development of the Vegan Diet Quality Index (VEGANScreener) for the European population [86]. This underscores the need for appropriately planned vegan diets to ensure adequate intake of nutrients typically absent or present in low bioavailability in plant-based foods. This need is particularly critical for ‘new vegans’, who often adopt less balanced diets with a higher risk of micronutrient deficiencies [88].

The consumption of plant-based meat analogues was not associated with an unhealthy vegan diet in the VEGANScreener, provided that their sodium and saturated fat content remained low. These findings suggest that the use of fats such as coconut and palm oil should be avoided in the formulation of such products, while the inclusion of vegetable oils rich in long-chain omega-3 fatty acids should be prioritized to support the overall nutritional quality of vegan diets [88].

All stakeholder groups agreed that the sensory quality of PBMAs is essential for their successful incorporation into the Brazilian diet. They also concurred that sensory attributes should be given equal importance to nutritional quality (recommendation 16); moreover, in situations where equal weighting is not feasible, sensory parameters should take precedence (recommendation 15).

With respect to innovation and technological aspects for PBMAs, the responses concerning the use of ingredients informed the development of recommendations that promote the utilization of ingredients derived from Brazilian biodiversity, encompassing all biomes (recommendation 17). The potential applications of these ingredients include alternative protein sources such as pulses and native fruits like castanha-do-brazil (*Bertholletia excelsa*), baru (*Dipteryx alata*), cashew nuts (*Anacardium occidentale*), and pequi (*Caryocar brasiliense*) highlighting opportunities for sustainable and culturally relevant product development [67,78,79,89,90,91,92,93].

Recommendation 18 did not reach consensus, receiving approval only from stakeholder group 1. This outcome suggests that some experts do not view consumer demand for products with fewer additives—commonly referred to as ‘clean label’—as a priority within the PBMA sector. However, it is noteworthy that a separate Delphi study aimed at identifying key innovations and challenges for the food industry by 2027 reported a 76.9% consensus regarding the growing demand for ‘natural foods,’ highlighting a possible disconnect between expert perceptions and broader market trends [43].

However, among the various sensory attributes, flavor emerges as the most critical factor influencing consumer acceptance of PBMAs [94,95]. Moreover, consumers tend to report higher sensory acceptability when these products mimic processed meat items—such as sausages, hamburgers, and breaded products—particularly in terms of flavor and/or texture [95]. In this context, the elimination of artificial additives, especially flavorings, poses a significant challenge to product development.

Clear labeling that specifies the protein source used in PBMAs, along with a reduced list of additives, has been shown to enhance consumer acceptance. Studies indicate that consumer perception linking PBMAs to highly processed foods and elevated sodium content can act as barriers to their consumption [96]. Therefore, regulatory frameworks play a crucial role in fostering consumer trust by promoting transparency and ensuring product integrity.

Recommendations 19, 22, and 23, which addressed regulatory aspects, reached consensus among the expert panel. In contrast, recommendations 20, 21, and 24 did not achieve consensus. It is noteworthy that none of the regulatory recommendations garnered unanimous agreement across all stakeholder groups, highlighting the complexity and multifaceted nature of the negotiations involved in establishing appropriate and effective regulatory frameworks for PBMAs.

Nonetheless, the consensus achieved reflects a favorable outlook regarding the establishment of a specific category encompassing all plant-based foods analogous to products of animal origin—such as meat, milk, eggs, fish, and honey (recommendation 19). Furthermore, experts agreed that the definition of ‘analogous food’ should be based primarily on the intended form of consumption (recommendation 22), rather than on the nutritional value or sensory characteristics of the products.

Regarding the inclusion of warning statements concerning the nature and nutritional value of PBMAs, consensus was reached solely for recommendation 23, relative to mandatory inclusion of the statement: “This product may have a different nutritional value than the corresponding animal-derived product” on PBMAs. The warning statement ‘This product does not replace its animal-based counterpart in nutritional or functional terms,’ included as recommendation 24 in the study, did not achieve consensus among the experts.

The regulation of PBMAs was considered a secondary priority in a separate consultation conducted with Brazilian experts aimed at identifying measures to promote the production and consumption of plant-based meat analogues [12]. Beyond pricing concerns, the experts highlighted that modifying the regulatory environment to ensure fair competition for alternative protein companies—such as implementing equitable labeling laws, removing subsidies for animal-based products, and adopting true-cost accounting—along with identifying new crops as viable sources of plant protein for PBMA production, were also viewed as secondary priorities [12].

### 3.5. Practical Implications

The formulation of nutritionally adequate plant-based meat analogues (PBMAs) significantly influences their sensory characteristics, technological performance, and production costs. However, the absence of standardized parameters for nutritional quality and product identity frequently hampers the food industry’s ability to use existing products as benchmarks during the development process. To address this regulatory gap, regulatory agencies worldwide have concentrated their efforts on developing guidelines for the labeling and nomenclature of plant-based meat analogues (PBMAs). In Brazil, public consultations regarding official regulatory documents were conducted in 2023. However, no significant progress has been made toward the establishment of clear identity standards for plant-based meat analogues (PBMAs) [97]. This same scenario is found in other countries, although there are discussions, no specific regulations on PBMA have been found. In the US, the FDA has released draft guidance for the industry to help consumers identify products; however, the draft provides specifications for nomenclature and labeling is not mandatory [98]. Consensus-based recommendations could help shape standards and serve as a valuable reference for regulatory bodies in the formulation of identity and quality criteria for PBMAs.

In the absence of formal standards, expert-driven approaches such as the Delphi method offer a valuable means of achieving consensus on the nutritional characteristics of healthy plant-based meat analogues (PBMAs). This, in turn, can effectively support and enhance the food industry’s research and development efforts. Notably, it underscores a broader challenge: the need to develop formulations that are not only technically feasible but also nutritionally adequate and aligned with consumer preferences.

The plant-based food sector has expanded in recent decades. The first generations used soy as the main ingredient, while new sources of protein emerged and new technologies accompanied this evolution, allowing for improvements in flavor, texture, and nutritional aspects. It is also worth noting that as technologies evolve and production increases, costs decrease, making food more accessible [99]. Thus, for the Brazilian industry, which is still in the first generation of plant-based products, following the consensus established by experts is necessary to enhance the foods produced in the country.

Incorporating nutritional recommendations aligned with established health guidelines into the formulation of plant-based meat analogues (PBMAs) can play a significant role in reducing the prevalence of chronic non-communicable diseases, particularly those associated with the consumption of conventional meat products. The World Health Organization (WHO), through its International Agency for Research on Cancer (IARC), has classified processed meats as Group 1 carcinogens, indicating that there is sufficient evidence to confirm their carcinogenicity in humans. Specifically, the daily consumption of a 50 g portion of processed meat is associated with an 18% increase in the risk of developing colorectal cancer. Additionally, the consumption of meat—particularly red meat—has been linked to a heightened risk of cardiovascular disease and type 2 diabetes.

This context underscores the opportunity of PBMAs to contribute to healthier diets. The partial or complete substitution of meat with plant-based foods has emerged as a major dietary trend over the past decade. While motivated in part by nutritional and health concerns, this shift is also driven by broader considerations, including environmental sustainability and animal welfare. Rather than representing a singular or niche movement, it reflects a broader rethinking of food systems and consumption patterns. Advances in technology, along with the development of innovative ingredients and additives, are expected to further stimulate the growth of the plant-based food market, particularly in the area of meat analogues.

To unlock the full potential of this transition, a holistic approach is needed as the health potential of plant-based diets depends not only on the composition of individual products, but also on the planning of the overall diet—ensuring that macro- and micronutrients are consumed in adequate amounts and bioavailable forms throughout the day. The expert-endorsed recommendations aim to support this broader nutritional strategy by guiding the development of PBMAs that align with public health priorities and dietary intake recommendations.

## 4. Conclusions

Following extensive collaboration with experts, we established 12 nutritional recommendations to guide the development of plant-based meat analogues with a composition profile aligned with a healthy diet. In addition, six other recommendations—addressing sensory, technological, and regulatory aspects—also achieved consensus among the experts.

The study established broad consensus among experts regarding the need to limit critical nutrients—particularly sodium and saturated fat—and to ensure minimum levels of protein and dietary fiber in PBMAs. These priorities reflect strong alignment with national and international dietary guidelines and suggest growing convergence between public health and product formulation goals.”

Conversely, there was no consensus on more ambitious targets such as higher levels of micronutrient fortification or omega-3 enrichment, likely due to feasibility concerns, cost implications, and knowledge gaps in the nutritional composition of currently available products.”

These findings reinforce the importance of developing evidence-based nutritional recommendations for plant-based meat analogues. While PBMAs can contribute meaningfully to nutrient intake, it is important to recognize that no single product can ensure dietary adequacy on its own.

## Figures and Tables

**Table 1:** Questions sent to experts in the first round of the Delphi study.

Nutritional Aspects	Questions
Proteins	1. What is your assessment of the protein adequacy in plant-based meat analogues, both in quantitative and qualitative terms? *(Your response may address aspects such as total protein content, amino acid composition, protein digestibility, and other relevant indicators of protein quality.)*
Fats	1. What is your assessment of the adequacy of fat content in plant-based meat analogues, considering both qualitative and quantitative aspects? *(Your response may address total fat content, levels of saturated, monounsaturated, polyunsaturated, and trans fats, as well as the overall fatty acid composition.)*
Carbohydrates	1. What is your assessment of the adequacy of carbohydrate content in plant-based meat analogues, both in qualitative and quantitative terms? *(Your response may address aspects such as total carbohydrates, soluble and insoluble dietary fiber, and other relevant carbohydrate-related parameters.)*
Sodium	What are your thoughts on the sodium content in plant-based meat analogues?
Vitamins and Minerals	1. How would you evaluate the adequacy of vitamins and minerals content in plant-based meat products, considering both qualitative and quantitative aspects? *(Your response may encompass B vitamins as well as fat-soluble vitamins (A, D, E, and K) and for minerals (Iron and Zinc) and may address factors such as bioaccessibility).*
**Sensory Aspects**	**Questions**
	1. Have you ever tasted vegetable products that are similar to meat products sold in Brazil?
	How would you evaluate the sensory quality of plant-based meat analogue products currently available on the Brazilian market?
	2. In your opinion, what are the primary sensory limitations or challenges observed in plant-based meat analogue products currently available on the market?
**Technological Aspects**	**Questions**
	1. Please list any guidelines you consider important regarding the use of ingredients in the formulation of plant-based products. *In your response, you may address aspects related to the selection and application of protein sources, lipid components, dietary fibers, and other functional or nutritional ingredients that are critical to product development.*
	2. In your opinion, should any specific ingredients or additives be restricted or prohibited in the formulation of plant-based meat products? *If so, please specify which ones and provide the rationale for their limitation or exclusion.*
**Innovative Aspects**	**Questions**
	1. In your opinion, what potential innovations could be applied in the development of plant-based meat products to address existing sensory and nutritional limitations?
	2. In the development of new plant-based meat products, which characteristics do you believe should be prioritized to enhance product quality and consumer acceptance?
**Regulatory Aspects**	**Questions**
	Plant-based products definition provided by Ministry of Agriculture and Livestock, the regulatory authority for animal and plant-based products in Brazil [1]: “A food product composed of raw materials of plant origin that bears a relationship to a corresponding animal-based product”. 1. Do you agree with the definition mentioned? Justify your answer.
	2. What expectations do you have regarding a product that includes the term ‘analogue’ in its sales name?

**Table 2:** Panel experts’ characteristics.

Characteristics	Round 1 (*n* = 10)	Round 2 (*n* = 20)	Round 3 (*n* = 34)
	*n* (%)	*n* (%)	*n* (%)
Stakeholder			
1. Researchers from institutes and/or universities	4 (40%)	10 (50%)	20 (58%)
2. Representatives from scientific societies, non-profit organizations, civil society, and government agencies	3 (30%)	5 (25%)	8 (24%)
3. Representatives from associations linked to the food industry	3 (30%)	5 (25%)	6 (18%)
Education background			
Graduate	0 (0%)	2 (10%)	6 (17%)
Postgraduate specialization	1 (10%)	2 (10%)	3 (9%)
Master’s degree	2 (20%)	4 (20%)	6 (17%)
PhD (doctorate)	5 (50%)	8 (40%)	9 (26%)
Post-doctoral	2 (20%)	4 (20%)	10 (29%)
Professional experience time			
1 to 5 years	1 (10%)	3 (15%)	3 (9%)
5 to 10 years	1 (10%)	3 (15%)	5 (15%)
10 to 15 years	1 (10%)	4 (20%)	4 (11%)
15 to 20 years	2 (20%)	2 (10%)	5 (15%)
More than 20 years	5 (50%)	8 (40%)	17 (50%)
Knowledge rating in a plant-based topic (1 to 10)			
Level 9	4 (40%)	7 (35%)	10 (30%)
Level 8	3 (30%)	6 (30%)	9 (26%)
Level 7	2 (20%)	4 (20%)	8 (23%)
Level 6	1 (10%)	3 (15%)	5 (15%)
Level 5	0 (0%)	0 (0%)	2 (6%)
Stated level of interest in the topic			
I have a strong interest in the topic, given its direct relevance to my professional practice.	7 (70%)	14 (70%)	24 (70%)
I am interested in the topic, as it is indirectly related to my professional practice.	3 (30%)	6 (30%)	10 (30%)

**Table 3:** Expert responses for open questions of Delphi round one.

Nutritional Aspects	Questions	Responses
Proteins	1. What is your assessment of the protein adequacy in plant-based meat analogues, both in quantitative and qualitative terms?	1. “Ensuring adequate protein content in plant-based analogues is generally more straightforward than achieving an optimal amino acid profile; nonetheless, it remains feasible. However, it is important to question whether consumers prioritize such nutritional characteristics or are primarily guided by the sensory attributes of these products. It should be emphasized that plant-based analogues are intended to complement a broader dietary pattern and are unlikely to serve as the sole source of protein for consumers. Although protein digestibility is a critical nutritional parameter, it remains largely inaccessible as information to the general public—not only in the context of meat analogues but also for food products more broadly.” 2. “I do not consider protein standardization to be necessary. Plant-based meat analogues are expected to have nutritional profiles that differ from those of conventional meat products, just as various meat products themselves can exhibit considerable nutritional variation. Therefore, aligning the protein composition of analogues to that of meat is not essential.” 3. “Establishing minimum parameters for protein quantity and amino acid quality, differentiated by type of analogue, could represent a viable strategy to address this gap. Such an approach is already mandated, for instance, in the context of nutritional claims for protein content according to Brazilian nutrition labeling law and is also employed as a compositional requirement for certain foods intended for special dietary purposes. However, the inclusion of digestibility as a criterion for protein quality remains overly complex, as evidenced by ANVISA’s (Brazilian Health Surveillance Agency) regulatory experience with foods targeted at athletes.” 4. “For meat analogue products, it is essential that their formulation is based on protein-rich matrices such as soy, wheat, and other legumes. From a quantitative perspective, while increased protein content may add market value, I do not believe that protein quantity and bioavailability parameters should mirror those used for animal-based products. In the context of a plant-based diet, protein intake is understood as a component of the overall dietary pattern rather than being concentrated in specific key items, as is typically the case with meat and eggs in omnivorous diets. Therefore, I advocate for a unified standard for plant-based meat analogues—one that ensures the use of vegetable protein sources while establishing a minimum protein content based on total protein per serving and the profile of essential amino acids, such as leucine. A useful reference for this standard could be the amino acid adequacy table outlined in Brazilian nutrition labeling law, which defines aminograms for protein-rich foods and could be adapted to the specific characteristics of plant-based proteins.”
Fats	1. What is your assessment of the adequacy of fat content in plant-based meat analogues, considering both qualitative and quantitative aspects?	1. “In general, plant-based analogues of protein-rich animal products tend to exhibit a more favorable lipid profile, primarily due to the use of vegetable oils and fats, which are typically lower in saturated fatty acids and higher in unsaturated fatty acids. As a result, the fat composition of these products is not typically considered a nutritional concern. It is important to note that consumers have access to information on total fat, saturated fat, and *trans* fat content, as these nutrients are mandatorily declared on nutrition labels in accordance with Brazilian nutrition labeling law. The amounts of monounsaturated and polyunsaturated fats may be declared voluntarily. Nutritional claims related to fat content are also permitted on a voluntary basis, provided that the product complies with the compositional and labeling requirements established by Brazilian nutrition labeling law. Notably, the use of partially hydrogenated oils—the primary source of industrial *trans* fats—is prohibited in food formulations, and the maximum allowable *trans* fatty acid content in refined vegetable oils is limited to 2%, as established by Brazilian food law. Therefore, the presence of *trans* fats in plant-based foods available in Brazil is not considered a significant public health concern. However, the authorization and regulation of plant-based analogues that replicate high-fat animal products require further consideration, particularly as they may fall under additional or separate regulatory frameworks.” 2. “In my assessment, fats represent a greater nutritional and technological challenge than proteins in the development of plant-based meat analogues. Unlike animal-derived meats, which naturally contain saturated fats that contribute to cohesion, texture, and lubricity, these functional lipids are absent in the protein matrices typically used in plant-based formulations. As a result, fats must be added to replicate the structural and sensory roles of animal fat, with palm fat often being the most accessible and technologically effective option. Despite its functional advantages, the widespread use of palm fat in ultra-processed plant-based products raises nutritional concerns due to its high saturated fat content. It is therefore essential to identify strategies that ensure the inclusion of fats—crucial from a culinary standpoint—without compromising the nutritional quality of the final product. In this context, the use of hydrocolloids presents a promising alternative for enhancing texture and matrix cohesion, potentially reducing the reliance on palm fat in an excessive manner.” 3. “Vegetable fats are generally considered nutritionally more favorable, and their incorporation into the formulation of plant-based analogues does not pose significant challenges from a nutritional standpoint.”
Carbohydrates	1. What is your assessment of the adequacy of carbohydrate content in plant-based meat analogues, both in qualitative and quantitative terms?	1. “I do not consider carbohydrate equivalence between plant-based meat analogues and conventional meat products to be necessary. Plant-based analogues may possess distinct nutritional profiles, just as various meat products differ among themselves. Therefore, aligning carbohydrate content is not essential.” 2. “In general, the technological formulation of plant-based analogues often relies on the combination of carbohydrates and vegetable fats, which contributes to the structural and sensory properties of these products. As a result, the presence of carbohydrates in such formulations is typically higher.” 3. “Plant-based analogues of protein-rich animal foods tend to have a higher carbohydrate content, particularly in terms of dietary fiber. This is attributed both to the intrinsic composition of the plant-derived ingredients and their functional roles in product development. Consumers are provided with information on carbohydrate content—including total sugars and added sugars—on the nutrition label, in accordance with Brazilian nutrition labeling law. Furthermore, the voluntary use of nutrition claims related to sugars is permitted, provided the product complies with the compositional and labeling criteria outlined in Brazilian nutrition labeling law. Nevertheless, it remains important to consider whether analogues of animal-based foods containing added sugars will be authorized under current regulatory frameworks, given that such products are already subject to specific legislation.”
Sodium	1. What are your thoughts on the sodium content in plant-based meat analogues?	1. “It is hoped that these products contain low levels per 100 g of critical nutrients and, consequently, do not receive a front-of-package warning label on their nutritional information”. 2. “I do not consider sodium adjustment to be necessary. Plant-based products designed to mimic meat may naturally present different nutritional profiles from their animal-based counterparts, just as nutritional variability exists among different types of meat products themselves. Consequently, standardizing sodium levels across these categories is unwarranted. Moreover, according to Brazilian nutrition labeling law, products containing sodium levels above the established threshold are required to display front-of-package warning labels. Thus, the implementation of a specific sodium standard for plant-based analogues is unnecessary”. 3. “Considering that the sodium content of foods is disclosed in the nutritional information panel, and that products with high sodium levels are required to display front-of-pack nutritional labeling in accordance with Brazilian nutrition labeling law, there appears to be no specific concern regarding the sodium content of these products in comparison to other processed foods”. 4. “In the context of the high sodium intake associated with the Western dietary pattern prevalent in Brazil, the elevated sodium content of plant-based analogues may further compromise the overall quality of a plant-based diet. Therefore, alternative formulation strategies are needed to ensure these products retain desirable flavor characteristics without excessive sodium content”.
Vitamins and Minerals	1. How would you evaluate the adequacy of vitamin and minerals content in plant-based meat products, considering both qualitative and quantitative aspects?	1. “Concerns regarding the nutritional implications of replacing animal-derived foods with analogous plant-based products suggest the potential necessity for mandatory enrichment of these products with selected micronutrients. This would involve establishing minimum and maximum allowable limits, as well as specifying authorized compounds, tailored to each product category. Alternatively, specific guidelines could be developed to regulate the voluntary enrichment of these products”. 2. “It is well established that, quantitatively, plant-based meat analogs contain lower levels of certain vitamins compared to their animal-based counterparts. Therefore, during the formulation of plant-based analogs, supplementation with bioactive forms of these vitamins is essential, in amounts equivalent to those found in the products they aim to replicate”. 3. “Vitamin supplementation in analogous products can help ensure nutritional equivalence; however, it is imperative to assess the stability of these added vitamins throughout the product’s shelf life and consumption.
**Sensory Aspects**		
	1. In your opinion, what are the primary sensory limitations or challenges observed in plant-based meat analogue products currently available on the market?	1. “It is difficult to generalize, as there is considerable variation among products currently available on the market. However, plant-based meat analogues often exhibit a saltier flavor profile. Additionally, some products lack the melt-in-the-mouth sensation typically associated with animal fat, which may affect overall sensory experience”. 2. “Residual flavors from protein concentrates and other ingredients, the use of overly artificial-tasting flavor maskers, and challenges in replicating the texture of solid products represent significant sensory limitations in the development of plant-based meat analogues”. 3. “Currently, there are no additives or processing aids authorized for use in foods classified as plant-based analogue products by Brazil’s Ministry of Agriculture, Livestock and Food Supply (MAPA). This regulatory gap poses a significant barrier to product formulation, given the essential role of various additives in enhancing the sensory attributes of processed foods. Consequently, one of the primary limitations identified in the proposed regulatory framework is the lack of approval for additives and adjuvants that are critical for achieving sensory characteristics comparable to those of animal-based products.”
**Technological Aspects**		
	1. Please list any guidelines you consider important regarding the use of ingredients in the formulation of plant-based products.	1.” Technological innovation in plant-based products should not be unduly restricted, as this is an emerging field in which premature regulatory limitations may hinder the development of novel technologies and ingredients. Such innovations have the potential to significantly enhance the sensory attributes, nutritional quality, and environmental sustainability of plant-based meat analogues.” 2. “The use of food additives and processing aids is governed by established principles in Brazilian health legislation. These principles stipulate that such substances must be proven safe for human consumption under their intended conditions of use, employed in the minimum effective quantities to achieve the desired technological function, and must not mislead consumers. Additionally, only those substances included in the positive lists defined by Brazilian health legislation—organized by food category and authorized by ANVISA—are permitted for use. At present, there are no additives specifically authorized for the food category encompassing plant-based analogue products. It is also important to consider that, depending on the nature of label claims associated with these products, the use of certain types or classes of additives may be deemed inappropriate”.
	2. In your opinion, should any specific ingredients or additives be restricted or prohibited in the formulation of plant-based meat products?	1. “Ingredients that pose safety risks or have the potential to mislead or confuse consumers—particularly in relation to the claims made on product labels—should not be permitted for use in food products”. 2. “I do not believe that any additive currently permitted for use in animal-based products within the same category should be prohibited in plant-based analogues”.
**Innovative Aspects**		
	1. In your opinion, what potential innovations could be applied in the development of plant-based meat products to address existing sensory and nutritional limitations?	1. “Exploration of underutilized plant-based ingredients and emerging technologies, such as precision fermentation, is essential for advancing innovation in the development of plant-based products”. 2. “The regulatory framework can be notably restrictive regarding formulation options for plant-based analogue products. Such limitations are likely to hinder the development of products with sensory and nutritional characteristics comparable to those of animal-based counterparts. Consequently, a comprehensive review of the proposed regulation is warranted”.
	2. In the development of new plant-based meat products, which characteristics do you believe should be prioritized to enhance product quality and consumer acceptance?	1. “Sustainability, along with improvements in texture and flavor, are critical factors for ensuring consumer acceptance and the successful integration of these products into the diet”. 2. “Sensory and nutritional characteristics”. 3. “Key considerations include product safety, nutritional quality, and regulatory compliance with labeling standards.” 4. “Striking a balance between nutritional quality and sensory attributes should be a central priority in the development of plant-based analogue products”.
**Regulatory Aspects**		
	Plant-based products definition: “A food product composed of raw materials of plant origin that bears a relationship to a corresponding animal-based product regulated by the Ministry of Agriculture and Livestock”. 1. Do you agree with the definition mentioned? Justify your answer.	1. “No. The proposal requires substantial improvement to ensure that the regulation of this emerging product category is both effective and proportionate in addressing the current challenges of the plant-based food market. This includes careful consideration of the compositional and presentation characteristics of these products, the potential for future innovations, existing regulations on food identity and quality standards, as well as relevant international regulatory frameworks. The main issues associated with this definition are: - The restriction of plant-based analogue products to formulations composed exclusively of ingredients of plant origin. This limitation fails to encompass the majority of plant-based products currently available on the market, which typically include plant-based protein foods designed to mimic animal-derived protein products. These formulations often incorporate ingredients from diverse origins—including animal, microbial (e.g., bacterial), and synthetic sources—to fulfill various technological, nutritional, and sensory functions. - These ingredients are incorporated into the formulation of plant-based foods to serve technological (e.g., additives), nutritional (e.g., nutrient source compounds), functional (e.g., probiotics), and sensory (e.g., salt) purposes. The imposed restriction significantly limits the potential for innovation within this category by prohibiting the inclusion of various novel foods and ingredients that may, in the near future, be approved as alternative protein sources—such as insects, cultured meat, and ingredients derived through nanotechnology or precision fermentation. Furthermore, the rationale for excluding ingredients that are considered safe and are widely used in the broader food industry remains unclear. This limitation adversely affects the scope, effectiveness, and proportionality of the proposed regulatory framework. It is also important to highlight that animal-based products are permitted to include ingredients of diverse origins in their formulations, including those that aim to replicate traditional animal-derived foods (e.g., dairy blends as alternatives to cream)”. 2. “I agree with the proposed definition, as it provides a clear and structured framework for categorizing plant-based analogue products while aligning with current scientific and regulatory standards”.
	2. What expectations do you have regarding a product that includes the term ‘analogue’ in its sales name?	1. “The term ‘vegetable analog of’ may not be easily understood by consumers, as it is not commonly used in the everyday vocabulary of Brazilians. Therefore, I suggest adopting a more consumer-friendly and transparent nomenclature, such as that already in use—for example, ‘burger made with (corresponding vegetable)”. 2. “That it exhibits sensory characteristics and functional performance equivalent to those of the corresponding animal-based product”. 3. “The term ‘analogous’ implies a product that is similar to another. Although this designation is qualified by the term ‘vegetable,’ consumers may interpret it as indicating that the product shares similar sensory and/or nutritional characteristics, or that it can be used in the same manner and for the same culinary purposes as its animal-based counterpart. However, based on the proposed compositional requirements, there is neither a guarantee nor, in some cases, a possibility of achieving nutritional equivalence or similarity in usage and preparation”.

**Table 4:** Recommendations evaluated in Delphi round 2 and agreement coefficient (AC) according to relevance and adequacy.

Recommendations	AC Relevance	AC Adequacy
Nutritional Aspects		
1. The minimum protein content in plant-based meat analogue products should be equivalent to that of conventional meat products, according to Brazilian Identity and Quality Standard. For example: the minimum protein content in a burger should be set at 15% [81].	80%	80%
2. The minimum protein content in plant-based meat analogue products should be 20% of Daily Value (%DV) equivalent to 10 g per serving size [62,63].	80%	85%
3. The selection of raw materials for the development of plant-based meat analogue products must ensure a composition of essential amino acids that meets the nutritional requirements of adults, in accordance with the standards set forth in Brazilian Food Labeling Law [60]. An amino acid composition is considered adequate when no limiting amino acids are present. The required amino acid profile per gram of protein is as follows: histidine–15 mg, isoleucine–30 mg, leucine–59 mg, lysine–45 mg, methionine + cysteine–22 mg, phenylalanine + tyrosine–38 mg, threonine–23 mg, tryptophan–6 mg, and valine–39 mg.	65%	60%
4. The selection of plant-based raw materials used to supply lipids in the formulation of plant-based meat analogue products should predominantly provide monounsaturated and polyunsaturated fatty acids.	85%	85%
5. The selection of plant-based raw materials intended to supply lipids in the formulation of plant-based meat analogue products should ensure the inclusion of omega-3 fatty acids, particularly at levels sufficient to qualify as a source of alpha-linolenic acid (ALA), defined as a minimum of 300 mg per serving size [62,63].	65%	65%
6. The selection of plant-based raw materials used to supply lipids in the formulation of plant-based meat analogue products should ensure the provision of polyunsaturated fatty acids, with an omega-6: omega-3 ratio not exceeding 10:1 [72].	70%	70%
7. The total fat content in plant-based meat analogue products should not exceed 30% DV for total energy value [52].	90%	90%
8. The saturated fat content in plant-based meat analogue products should not exceed 10% DV for total energy value [52].	90%	90%
9. The saturated fat content in plant-based meat analogue products should remain below the threshold established for the application of front-of-package nutritional warning labels for solid and semi-solid foods, as defined by Brazilian Food Labeling Law (equivalent to 6 g per 100 g) [62,63].	90%	90%
10. Plant-based meat analogue products must contain dietary fiber levels that meet at least the minimum requirement to be classified as a source of fiber, as defined by Brazilian Food Labeling Law (equivalent to 10% DV, or 2.5 g of fiber per serving size) [62,63].	80%	85%
11. Plant-based meat analogue products must contain dietary fiber levels that meet at least the minimum requirement to be classified as a high content of fiber, as defined by Brazilian Food Labeling Law (equivalent to 20% DV, or 5.0 g of fiber per serving size) [62,63].	45% *	50% *
12. The sodium content in plant-based meat analogue products must be below the threshold established for front-of-package nutritional warning labeling for solid and semi-solid foods, in accordance with Brazilian Food Labeling Law, which sets the limit at 600 mg per 100 g [62,63].	90%	85%
13. The sodium content in plant-based meat analogue products should be less than 1 mg per kilocalorie, in accordance with PAHO/WHO nutritional guidelines [65].	65%	60%
14. Plant-based meat analogue products should be fortified with minerals (iron and zinc) and B vitamins (B6 and B12) at levels sufficient to qualify as sources of these nutrients, in accordance with Brazilian Food Labeling Law [62,63], which defines this threshold as 15% of DV per serving size.	75%	80%
15. Plant-based meat analogue products should be fortified with minerals (iron and zinc) and B vitamins (B6 and B12) at levels sufficient to qualify as sources of these nutrients, in accordance with Brazilian Food Labeling Law [62,63], which defines this threshold as 30% of DV per serving size.	65%	65%
Sensory		
16. When the objective is to replicate meat products, the sensory attributes of plant-based analogues should be prioritized over nutritional aspects.	65%	65%
17. When the objective is to replicate meat products, the nutritional quality of plant-based analogues should take precedence over sensory.	45% *	50% *
18. When the objective is to replicate meat products, sensory and nutritional attributes should be given equal importance to effectively achieve product equivalence.	85%	85%
Technology		
19. Plant-based meat analogue products should be recognized as a distinct food category within legislation governing the use of food additives and processing aids.	85%	85%
Regulatory		
20. A food product formulated from plant-based raw materials that exhibits sensory characteristics comparable to its corresponding animal-derived product regulated by the Ministry of Agriculture and Livestock.	92%	92%
21. A food product formulated from plant-based raw materials that demonstrates nutritional equivalence to the corresponding animal-derived product regulated by the Ministry of Agriculture and Livestock.	75%	75%
22. A food product formulated from plant-based raw materials that corresponds to the consumption format of the analogous animal-derived product regulated by the Ministry of Agriculture and Livestock.	97%	97%
23. Mandatory inclusion of the statement: “This product may have a different nutritional value than the corresponding animal-derived product” on plant-based analogue products.	85%	85%
24. Mandatory inclusion of the statement: “This product does not replace its animal-based counterpart in nutritional or functional terms” on plant-based analogue products.	65%	65%

* Recommendation excluded.

**Table 5:** Recommendations evaluated in Delphi round 3 according to relevance, feasibility and total agreement.

Recommendations	Relevance (Mean ± SD)	Feasibility (Mean ± SD)	Total Agreement (Mean ± SD)	Consensus (%)
Nutritional Aspects				
1. The minimum protein content in plant-based meat analogue products should be equivalent to that of conventional meat products, according to Brazilian Identity and Quality Standard. For example: the minimum protein content in a burger should be set at 15% [73].	8.06 ± 1.92	7.50 ± 2.48.	7.78 ± 1.81	82% all stakeholders
2. The minimum protein content in plant-based meat analogue products should be 20% of Daily Value (%DV) equivalent to 10 g per serving size [60].	7.00 ± 2.62	6.93 ± 1.41	6.96 ± 2.31	67% all stakeholders
3. The selection of raw materials for the development of plant-based meat analogue products must ensure a composition of essential amino acids that meets the nutritional requirements of adults, in accordance with the standards set forth in Brazilian Food Labeling Law [60]. An amino acid composition is considered adequate when no limiting amino acids are present. The required amino acid profile per gram of protein is as follows: histidine–15 mg, isoleucine–30 mg, leucine–59 mg, lysine–45 mg, methionine + cysteine–22 mg, phenylalanine + tyrosine–38 mg, threonine–23 mg, tryptophan–6 mg, and valine–39 mg.	7.24 ± 2.34	6.69 ± 2.32	6.69 ± 2.13	73% stakeholder 1
4. The selection of plant-based raw materials used to supply lipids in the formulation of plant-based meat analogue products should predominantly provide monounsaturated and polyunsaturated fatty acids.	7.57 ± 2.50	7.60 ± 1.85	7.59 ± 1.97	76% all stakeholders
5. The selection of plant-based raw materials intended to supply lipids in the formulation of plant-based meat analogue products should ensure the inclusion of omega-3 fatty acids, particularly at levels sufficient to qualify as a source of alpha-linolenic acid (ALA), defined as a minimum of 300 mg per serving size.	5.69 ± 2.83	5.75 ± 2.71	5.72 ± 2.48	50% * stakeholder 1
6. The selection of plant-based raw materials used to supply lipids in the formulation of plant-based meat analogue products should ensure the provision of polyunsaturated fatty acids, with an omega-6: omega-3 ratio not exceeding 10:1 [56].	6.57 ± 2.63	6.33 ± 2.44	6.45 ± 2.25	60% stakeholders 1 and 2
7. The total fat content in plant-based meat analogue products should not exceed 30% DV for total energy value [50,55].	7.48 ± 2.80	7.66 ± 2.49	7.57 ± 2.49	73% all stakeholders
8. The saturated fat content in plant-based meat analogue products should not exceed 10% DV for total energy value [50,54,55].	8.33 ± 2.58	7.81 ± 2.29	8.05 ± 2.27	76% all stakeholders
9. The saturated fat content in plant-based meat analogue products should remain below the threshold established for the application of front-of-package nutritional warning labels for solid and semi-solid foods, as defined by Brazilian Food Labeling Law (equivalent to 6 g per 100 g) [60].	8.12 ± 2.42	7.90 ± 1.86	8.01 ± 1.91	79% all stakeholders
10. Plant-based meat analogue products must contain dietary fiber levels that meet at least the minimum requirement to be classified as a source of fiber, as defined by Brazilian Food Labeling Law (equivalent to 10% DV, or 2.5 g of fiber per serving size) [60].	6.75 ± 2.78	7.75 ± 2.12	7.25 ± 2.33	73% stakeholders 1 and 2
11. The sodium content in plant-based meat analogue products must be below the threshold established for front-of-package nutritional warning labeling for solid and semi-solid foods, in accordance with Brazilian Food Labeling Law, which sets the limit at 600 mg per 100 g [60].	8.00 ± 2.88	7.27 ± 2.15	7.86 ± 2.39	76% all stakeholders
12. The sodium content in plant-based meat analogue products should be less than 1 mg per kilocalorie, in accordance with PAHO/WHO nutritional guidelines [50]	6.72 ± 2.85	6.51 ± 2.62	6.62 ± 2.55	70% stakeholders 1 and 3
13. Plant-based meat analogue products should be fortified with minerals (iron and zinc) and B vitamins (B6 and B12) at levels sufficient to qualify as sources of these nutrients, in accordance with Brazilian Food Labeling Law [60] which defines this threshold as 15% of DV per serving size.	6.50 ± 2.90	7.00 ± 2.62	6.74 ± 2.42	64% stakeholders 1 and 2
14. Plant-based meat analogue products should be fortified with minerals (iron and zinc) and B vitamins (B6 and B12) at levels sufficient to qualify as sources of these nutrients, in accordance with Brazilian Food Labeling Law [60] which defines this threshold as 30% of DV per serving size.	5.45 ± 2.92	6.39 ± 2.99	5.92 ± 2.73	53% * stakeholder 1
Sensory Aspects				
15. When the objective is to replicate meat products the sensory parameters of plant-based analogues should take precedence over nutritional quality.	7.12 ± 2.64	7.12 ± 2.57	7.24 ± 2.30	73% all stakeholders
16. When the objective is to replicate meat products the sensory and nutritional attributes should be given equal importance to effectively achieve product equivalence.	8.09 ± 1.81	7.27 ± 1.62	7.68 ± 1.51	82% all stakeholders
Technology Aspects				
17. The use of alternative proteins derived from Brazil’s rich biodiversity—including resources from various biomes and marine environments—should be prioritized in the development of plant-based products designed to mimic meat.	7.69 ± 2.67	7.24 ± 2.30	7.46 ± 2.39	80% all stakeholders
18. Formulations of plant-based meat products should aim to limit or avoid the inclusion of artificial additives and technological adjuvants, promoting cleaner and more natural ingredient profiles.	6.90 ± 3.07	6.15 ± 2.75	6.55 ± 2.76	67% * stakeholder 1
Regulatory Aspects				
19. Plant-based meat analogue products should be recognized as a distinct food category within legislation governing the use of food additives and processing aids.	7.30 ± 3.21	7.45 ± 2.68	7.30 ± 2.84	71% stakeholders 1 and 3
20. A food product formulated from plant-based raw materials that exhibits sensory characteristics comparable to its corresponding animal-derived product regulated by the Ministry of Agriculture and Livestock.	7.03 ± 2.92	6.72 ± 2.97	6.87 ± 2.87	65% * stakeholder 1
21. A food product formulated from plant-based raw materials that demonstrates nutritional equivalence to the corresponding animal-derived product regulated by the Ministry of Agriculture and Livestock.	6.60 ± 2.76	6.30 ± 2.82	6.45 ± 2.73	56% * stakeholders 1 and 2
22. A food product formulated from plant-based raw materials that corresponds to the consumption format of the analogous animal-derived product regulated by the Ministry of Agriculture and Livestock.	7.30 ± 2.98	7.54 ± 2.73	7.37 ± 2.84	70% stakeholders 1 and 3
23. Mandatory inclusion of the statement: “This product may have a different nutritional value than the corresponding animal-derived product” on plant-based analogue products.	7.33 ± 3.13	7.81 ± 3.13	7.57 ± 2.63	79% stakeholders 1 and 3
24. Mandatory inclusion of the statement: “This product does not replace its animal-based counterpart in nutritional or functional terms” on plant-based analogue products.	5.21 ± 3.25	5.93 ± 3.31	5.75 ± 3.12	41% * stakeholder 1

* Recommendations Lacking Consensus in the Delphi Study. Stakeholder 1: researchers from institutes and/or universities engaged in the development of PBMAs, or conducting research in the fields of alternative proteins, protein chemistry, and/or food nutritional value; Stakeholder 2: representatives from scientific societies, non-profit organizations, civil society, and government agencies; and Stakeholder 3: representatives from associations linked to the food industry that produce PBMAs. SD: Standard Deviation.

## Data Availability

The original contributions presented in this study are included in the article material. Further inquiries can be directed to the corresponding author.
